# Large-scale synthesis of hybrid metal oxides through metal redox mechanism for high-performance pseudocapacitors

**DOI:** 10.1038/srep20021

**Published:** 2016-01-25

**Authors:** Zhonghua Ren, Jianpeng Li, Yaqi Ren, Shuguang Wang, Yejun Qiu, Jie Yu

**Affiliations:** 1 Shenzhen Engineering Lab for Supercapacitor Materials, Shenzhen Key Laboratory for Advanced Materials, and Department of Material Science and Engineering, Shenzhen Graduate School, Harbin Institute of Technology, University Town, Shenzhen 518055, China

## Abstract

Electrochemical performance and production cost are the main concerns for the practical application of supercapacitors. Here we report a simple and universally applicable method to prepare hybrid metal oxides by metal redox reaction utilizing the inherent reducibility of metals and oxidbility of 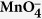 for the first time. As an example, Ni(OH)_2_/MnO_2_ hybrid nanosheets (NMNSs) are grown for supercapacitor application by self-reaction of Ni foam substrates in KMnO_4_ solution at room temperature. The obtained hybrid nanosheets exhibit high specific capacitance (2,937 F g^−1^). The assembled solid-state asymmetric pseudocapacitors possess ultrahigh energy density of 91.13 Wh kg^−1^ (at the power density of 750 W kg^−1^) and extraordinary cycling stability with 92.28% capacitance retention after 25,000 cycles. Co(OH)_2_/MnO_2_ and Fe_2_O_3_/MnO_2_ hybrid oxides are also synthesized through this metal redox mechanism. This green and low-cost method is capable of large-scale production and one-step preparation of the electrodes, holding promise for practical application of high-performance pseudocapacitors.

In terms of the charge storage mechanism supercapacitors can be classified into electrochemical double layer capacitors (EDLCs) and pseudocapacitors. The EDLCs, which usually use carbon materials such as nanoporous carbon derived from metal-organic frameworks^1^,^2^ and graphene^3^ as the electrode materials, possess high power capability and good stability. Recently, pseudocapacitors have attracted special attention owing to the capability for storing much more electrical energy than carbon based EDLCs^4^,^5^. In recent years, considerable efforts have been devoted to explore high-performance pseudocapacitive materials owing to their high theoretical specific capacitances^6^,^7^,^8^. Among various pseudocapacitive materials, transition metal oxides/hydroxides such as RuO_2_, MnO_x_, NiO, Ni(OH)_2_, CoO_x_, Co(OH)_2_, VO_x_, and FeO_x_ have exhibited attractive performance for supercapacitor application^9^,^10^,^11^,^12^,^13^,^14^,^15^,^16^. The typical methods preparing the pseudocapacitive oxides include hydrothermal synthesis^12^,^16^,^17^,^18^,^19^,^20^,^21^, electrodeposition^9^,^15^,^22^,^23^,^24^, and solution-phase synthesis^10^,^11^,^14^,^25^,^26^,^27^,^28^. The hydrothermal synthesis is generally based on chemical reactions involving the reagents containing corresponding metal element at high temperature and pressure while the electrodeposition proceeds through potential-assisted redox reaction of metal salts. For these two methods, the productivity or the size (on substrates) of the products are mainly limited by the required equipment. Comparing with the hydrothermal synthesis and electrodeposition, solution-phase synthesis is simple, low-cost, and capable of large-scale production. Generally, the solution-phase synthesis relies on room temperature chemical reaction driven by the free energy changes or difference in redox potential. For instance, for the preparation of Ni(OH)_2_ and Co(OH)_2_, Ni and Co salts are used based on the chemical reaction of M^x+^ + xOH^−^ = M(OH)_x_ (M = Ni, Co) under basic conditions^14^,^26^. With respect to MnO_2_, various reducing agents are utilized such as manganese sulfate^10^, potassium borohydride^29^, sodium hypophosphite^29^, hydrochloric acid^29^, ethylene glycol^30^, and carbon^13^,^31^,^32^ to reduce KMnO_4_. Among these reducing agents, carbon is widely used following equation (1)^13^,^31^,^32^:


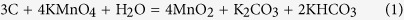


Analogous to this redox reaction, zerovalent metals should be able to reduce 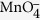 due to the inherent reducibility of metals^33^ and the oxidbility of 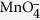. But dissimilarly, the products may not be pure MnO_2_ because the simultaneously produced metal oxide or hydroxide is insoluble. Therefore, this may be a new avenue to prepare hybrid metal oxides. It has been reported that the compounding of different metal oxides may generate synergistic and complementary effects for supercapacitor application, obtaining enhanced and versatile properties^13^,^18^,^34^. However, to the best of our knowledge, the preparation of hybrid metal oxides by this route has not been reported to date.

It is well known that the oxidbility of 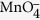 and product type are dependent on the solution acidity. According to the table of standard electrode potentials^35^, in neutral or weakly basic solutions the half and overall reactions of zerovalent metal (M) oxidation by 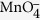 can be described as equations (2), (3), and (4):


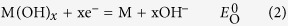



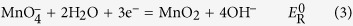



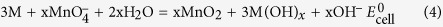


The occurring possibility of the reactions is determined by 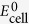 (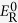 − 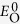). If 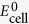 > 0, the redox reaction occurs spontaneously in the forward direction under standard conditions^36^. Therefore, it is reasonable to believe that different M(OH)_x_/MnO_2_ hybrids can be prepared by this simple reaction as long as 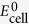 > 0. Taking Ni as an example, the overall reaction is described as equation (5):


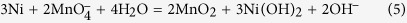



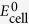
 value of equation (5) is calculated to be 1.315 V (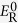 − 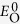 = 0.595 V − (−0.72 V)), indicating that this redox reaction occurs spontaneously^35^,^36^. During recent intensive research, it has been found that the oxides of many metals such as Mo, W, Zn, Bi, Cu, Sn, Ti, Ir, Pb, and La possess interesting electrochemical properties for energy storage^28^,^37^,^38^,^39^,^40^,^41^,^42^,^43^,^44^,^45^. According to the standard electrode potentials shown in Supplementary Table S1, these metals are capable of reducing 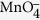 to produce corresponding hybrid metal oxides theoretically^36^. Consequently, this route is expected to be universally applicable for synthesizing multi-elemental oxides.

Herein, according to this idea, we established a novel process to prepare hybrid metal oxides. As a proof of concept, NMNSs were synthesized following equation (5) for supercapacitor application, where Ni foam was used as the reducing agent. Ni foam was chosen because it is one of the most widely used current collectors for energy storage devices due to its three-dimensional porous structure, high corrosion resistance, high electrical conductivity, and low cost^22^. In this work, therefore, Ni foam played dual roles of reducing agent and current collector. The NMNSs are directly grown on the Ni foam current collectors, enabling one-step preparation of supercapacitor electrodes without using any binders or conductive additives. Very recently, two-dimensional (2D) nanomaterials, particularly ultrathin layered nanosheet materials such as graphene and transition metal dichalcogenides (TMDs) have received increasing research interest owing to their distinctive and charming physical, chemical, and electronic properties for great potential applications in energy storage and conversion, catalysis, field-effect transistor (FET), sensors, and drug delivery, and so on^46^,^47^. For supercapacitor application, in particular, the ultrathin 2D nanostructure endows the active electrode materials with a high proportion of surface atoms and active sites on the exposed surfaces and sufficient contact with the electrolyte, short ion and electron diffusion path, benefiting fast charge transfer and electrochemical reactions. The present reaction described in this paper can not only proceed at room temperature without requiring any special equipment but also bring an ultrathin nanosheet structure. These advantages render it an industrially feasible and promising strategy for large-scale production of high performance pseudocapacitive materials.

## Results

### Preparation, characterization, and growth mechanism of NMNSs

**Figure 1:**
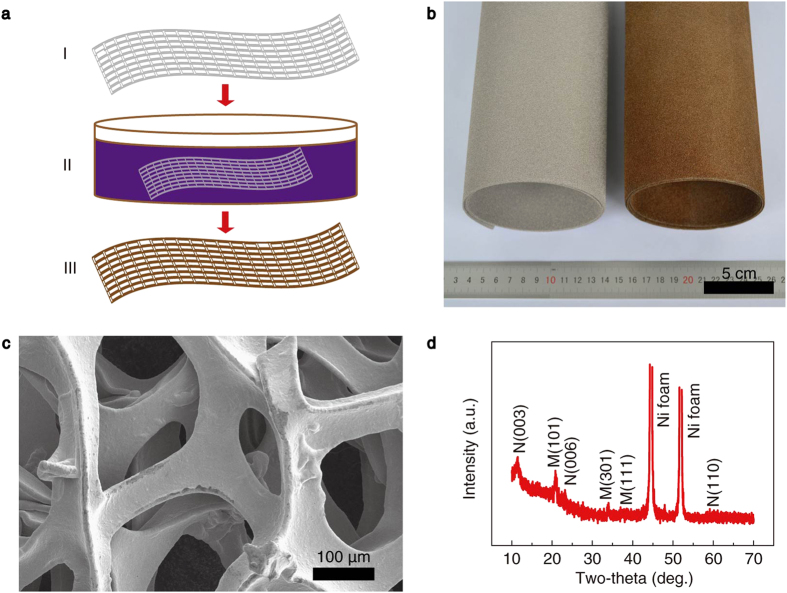
Preparation and characterization of the NMNSs. (**a**) A schematic diagram showing the preparation procedure. I, Pristine Ni foam; II, Redox reaction in KMnO_4_ solution; III, NMNSs coated Ni foam. (**b**) An optical image of the Ni foam before (left) and after (right) growth reaction. (**c**) SEM image of the Ni foam after the “soaking” process. (**d**) A typical XRD pattern (N and M represent Ni(OH)_2_ and MnO_2_, respectively).

The preparation process is schematically shown in Fig. 1a. The NMNSs were prepared by soaking a commercially available Ni foam in KMnO_4_ solution, after which the Ni foam substrates turned brown (Fig. 1b). It is worth mentioning that we carried out the experiments in darkness to eliminate the effect of KMnO_4_ decomposition under light illumination. By this really simple “soaking” process we have prepared binder-free electrodes as large as 25 × 100 cm^2^ (Fig. 1b and Supplementary Fig. S1), which are suitable for the fabrication of large-area and flexible supercapacitors. To the best of our knowledge, this electrode area is the largest among the previously reported electrode materials (Supplementary Table S2). Owing to the participation of the substrate element in the reaction, the anchoring strength of the nanosheets on Ni foam is very high, which was proved by an adhesion test (Supplementary Fig. S2). It is observed that the nanosheet film keeps intact after peeling a 3M scotch tape sticking on it, where no color changes are observed for both the film and the peeled tape. As shown in the scanning electron microscopy (SEM) image (Fig. 1c), a continuous film has been successfully grown on the Ni foam surface uniformly after the “soaking” process. Figure 1d shows the typical X-ray diffraction (XRD) pattern of the obtained samples. It is clear that both Ni(OH)_2_ and MnO_2_ peaks appear on the pattern. Except for the peaks originating from the Ni foam (JCPDS No. 04-0850), all the discernible peaks can be indexed to α-Ni(OH)_2_ (JCPDS No. 38-0715)^18^ and ramsdellite MnO_2_ (JCPDS No. 42-1316), implying coexistence of Ni(OH)_2_ and MnO_2_.

**Figure 2:**
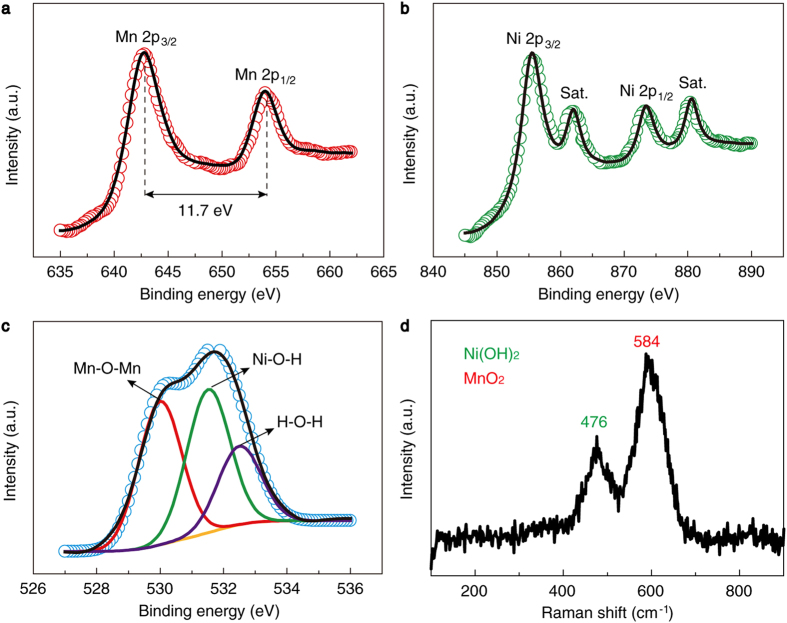
XPS and Raman spectra of the NMNSs. (**a**–**c**) XPS spectra of Mn 2p (**a**), Ni 2p (**b**), and O 1 s (**c**). (**d**) Raman spectrum.

X-ray photoelectron spectroscopy (XPS) and Raman measurements were performed to further determine the chemical composition and bond type of the products. The XPS survey spectrum indicates that the samples contain four elements, i.e., Mn, Ni, O, and C (Supplementary Fig. S3). The C element should be from the ambient contamination. Figure 2a presents the Mn 2p spectrum of the samples. The binding energies of Mn 2p_3/2_ and Mn 2p_1/2_ are centred at 642.4 and 654.1 eV, respectively, with a spin-energy separation of 11.7 eV, which is in good agreement with the previous reports for MnO_2_^18^. In Fig. 2b, the peaks of Ni 2p_3/2_ at 855.6 eV and Ni 2p_1/2_ at 873.4 eV indicate the presence of Ni(OH)_2_^18^,^48^. Figure 2c shows the O 1s spectrum, which displays three peaks at 529.9, 531.6, and 532.5 eV, being characteristic of the oxygen in oxides (Mn-O-Mn)^49^, hydroxides (Ni-O-H)^34^, and bound water (H-O-H)^34^,^50^, respectively. The atomic ratio of Ni to Mn calculated from the XPS spectra is 1.51:1, which is close to a ratio of 3:2. The typical Raman spectrum is shown in Fig. 2d. The bands at 476 and 584 cm^−1^ are assigned to the isostructural NiO_2_ units in Ni(OH)_2_^51^ and the symmetric stretching vibration (Mn-O) of the MnO_6_ octehedra^52^, respectively. Based on the above results, we verify that the chemical reaction for growing the hybrid nanosheets proceeds following equation (5).

**Figure 3:**
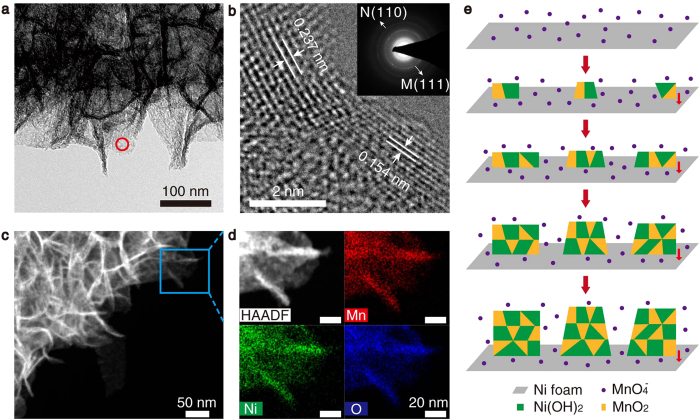
Morphology, composition, and formation mechanism of the NMNSs. (**a**) TEM image. The circle-marked area is the region for EDX test. (**b**) HRTEM image taken from the edge of a nanosheet. Inset: SAED image, N and M represent Ni(OH)_2_ and MnO_2_, respectively. (**c**) High magnification HAADF-STEM image. (**d**) Enlarged HAADF-STEM image of the rectangular region in panel c for EDX mapping and EDX mapping images of Mn, Ni, and O. (**e**) Possible growth mechanism of the NMNSs on Ni foam.

SEM, Transmission electron microscopy (TEM), and high-angle annular dark field scanning transmission electron microscopy (HAADF-STEM) images (Supplementary Fig. S4 and Fig. 3a,c) clearly show that the film is composed of ultrathin nanosheets with thickness of 2–7 nm, which are vertically aligned on the Ni foam substrates, interconnecting to form a porous structure. Energy-dispersive X-ray (EDX) spectra (Supplementary Fig. S5) taken on a single nanosheet (circle-marked area in Fig. 3a) indicates that each nanosheet contains Mn, Ni, O, and K elements. The high resolution TEM (HRTEM) image shown in Fig. 2b clearly reveals that two crystalline domains with different lattice fringe spacings coexist in the individual nanosheet. The lattice fringe spacings were measured to be 0.237 and 0.154 nm for the two domains, which correspond to the (111) plane of MnO_2_ and (110) plane of Ni(OH)_2_, respectively. The selected-area electron diffraction (SAED) pattern (inset in Fig. 3b) taken from a single nanosheet shows observable diffraction rings corresponding to the (111) plane of MnO_2_ and (110) plane of Ni(OH)_2_, respectively, consistent with the XRD pattern and HRTEM image. The EDX mapping images shown in Fig. 3d taken from the rectangular region in Fig. 3c indicate that Ni, Mn, and O are uniformly distributed in the nanosheets. These results confirm that Ni(OH)_2_ and MnO_2_ coexist in individual nanosheets in the form of small domains rather than forming different nanosheets, which may result from the unique growth mechanism of the NMNSs. Figure 3e presents the possible growth mechanism of the NMNSs on Ni foam. Because the reactant Ni is solid and immobile, the growth reaction can only occur on the surface of the Ni foam substrates and the Ni foam/nanosheets interfaces, resulting in a downward growth process at the nanosheet bottom. According to equation (5), Ni(OH)_2_ and MnO_2_ are produced simultaneously at the same site, which will crystallize *in situ* to form different phase domains adjacent to each other. By successive nucleation and growth, the nanosheets composed of Ni(OH)_2_ and MnO_2_ domains are obtained. These small domains form a large number of interfaces, providing numerous active sites for ion adsorption and insertion.

### Electrochemical characterization of NMNSs

**Figure 4:**
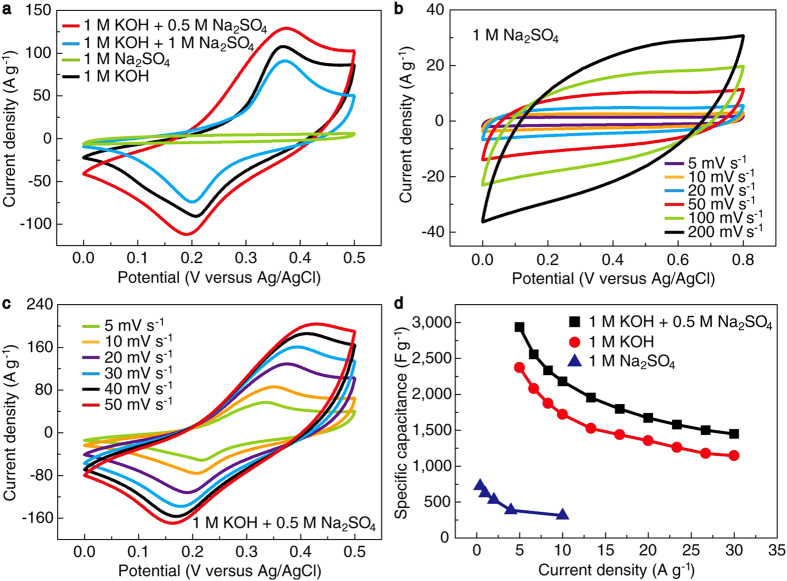
Electrochemical characterization of the NMNSs. (**a**) CV curves in a potential range of 0–0.5 V at a scan rate of 20 mV s^−1^ in different electrolytes. (**b**) CV curves in a potential range of 0–0.8 V at different scan rates in pure 1 M Na_2_SO_4_ electrolyte. (**c**) CV curves in a potential range of 0–0.5 V at different scan rates in the mixed electrolyte (1 M KOH + 0.5 M Na_2_SO_4_). (**d**) Specific capacitances calculated at various current densities in pure 1 M KOH, pure 1 M Na_2_SO_4_ and the mixed electrolyte (1 M KOH + 0.5 M Na_2_SO_4_).

Owing to the coexistence of Ni(OH)_2_ and MnO_2_ phases in the nanosheets, pure solutions of KOH and Na_2_SO_4_ and their mixed solutions with different concentrations and proportions were used as the electrolytes to characterize their electrochemical performance in this work. Figure 4a shows the cyclic voltammogram (CV) curves of the samples measured in a potential range of 0–0.5 V at a scan rate of 20 mV s^−1^ in different electrolytes. It is observed that the electrodes exhibit much higher current responses in KOH-contained solutions than in pure Na_2_SO_4_ solution. This is because the intrinsic Faradaic reaction current of Ni(OH)_2_ in KOH is much higher than that of MnO_2_ in Na_2_SO_4_^53^,^54^. Except in pure 1 M Na_2_SO_4_ solution, all other CV curves show similar shapes with two obvious symmetric peaks appearing in the negative and positive scan, implying the excellent reversibility of the Faradaic reactions between Ni^2+^/Ni^3+^ and OH^−^ 34,55. Interestingly, the CV curves at different scan rates in pure Na_2_SO_4_ solution exhibit a quasi-rectangular shape and near mirror-image symmetry in a wider potential range of 0–0.8 V (Fig. 4b), reflecting the typical electrochemical features of MnO_2_ as reported previously^24^. The corresponding galvanostatic charge/discharge (GCD) curves present nearly triangular shape with good linearity and symmetry and negligible internal resistance (IR) drop (Supplementary Fig. S6). The shapes of the CV and GCD curves suggest a rapid and reversible Faradaic reaction between Na^+^/H^+^ and MnO_2_ during the charge/discharge process^5^. With introducing Na_2_SO_4_ into the 1 M KOH solution to form a mixed solution of 1 M KOH + 0.5 M Na_2_SO_4_, the current response increases. The enclosed area of the CV curve is ~20% larger than that in pure 1 M KOH solution, implying a capacitance increase of ~20%. This may be because the size of Na^+^ is smaller than K^+^ and Na^+^ can get access to most of the MnO_2_ domains more easily, which improves the MnO_2_ utilization and thus the specific capacitance of the NMNSs. Correspondingly, the GCD curve in 1 M KOH + 0.5 M Na_2_SO_4_ solution shows a longer discharge time than in pure 1 M KOH solution (Supplementary Fig. S7). It is noted that with increasing the Na_2_SO_4_ concentration to 1 M in the mixed solution, the CV current decreases. In this case, excess Na^+^ causes aggregation and slows the ion diffusion, which lowers the ion adsorption efficiency^56^. The CV curves at different scan rates (Fig. 4c) and GCD curves at different current densities (Supplementary Fig. S8) were also measured in the mixed solution of 1 M KOH + 0.5 M Na_2_SO_4_. The voltage plateaus in the GCD curves well match the peaks in the CV curves. As shown in Fig. 4d, the maximum specific capacitance (based on the mass of NMNSs) calculated from the GCD curves in the mixed electrolyte of 1 M KOH + 0.5 M Na_2_SO_4_ reaches 2,937 F g^−1^ at 5 A g^−1^. Additionally, the specific capacitances of the NMNSs in pure KOH and Na_2_SO_4_ electrolytes were also calculated, which reach 2,325 F g^−1^ at 5 A g^−1^ and 723 F g^−1^ at 0.4 A g^−1^, respectively. These values are comparable and even higher than the previously reported Ni(OH)_2_ and MnO_2_ based electrode materials^10^,^11^,^12^,^13^,^18^,^20^,^21^,^23^,^26^,^37^,^55^,^57^,^58^,^59^,^60^,^61^,^62^ (Supplementary Table S3). Based on these results it is confirmed that the capacitance increase in the mixed electrolytes originates from the superposition contribution of MnO_2_. It can be seen that the NMNSs prepared by this simple “soaking” technique possess excellent electrochemical performance besides the advantage of process simplicity.

### Electrochemical characterization of solid-state asymmetric pseudocapacitors

**Figure 5:**
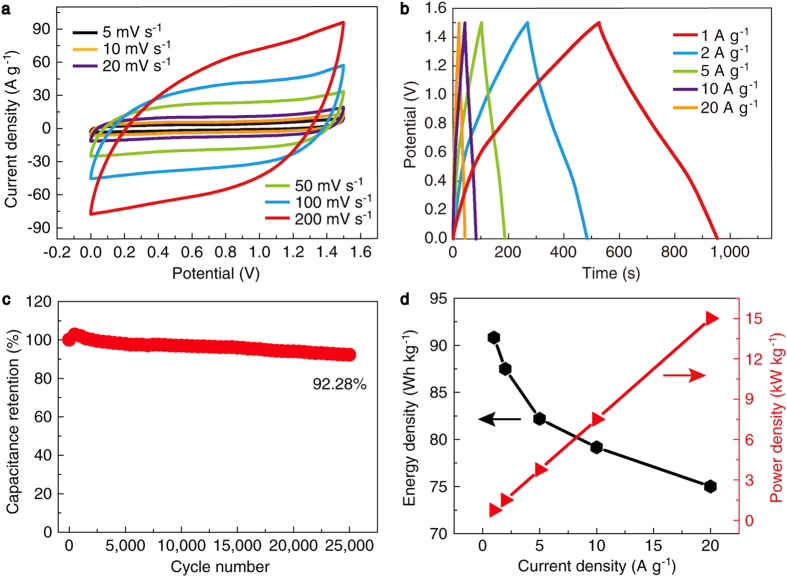
Electrochemical characterization of the asymmetric solid-state pseudocapacitors. (**a**) CV curves in a potential range of 0–1.5 V at various scan rates. (**b**) GCD curves at different current densities. (**c**) Cycling stability tested at a current density of 2 A g^−1^. (**d**) Energy and power densities at different current densities.

Solid-state asymmetric pseudocapacitors were assembled to evaluate the application potential of our materials. We used the NMNSs coated Ni foams and activated carbon (AC) as the cathode and anode respectively. As one of the most widely used solid-state electrolytes for supercapacitors, PVA/KOH gel electrolyte was chosen here as an example. Figure 5a shows the typical CV curves of the assembled pseudocapacitors in a voltage window of 1.5 V at different scan rates. The quasi-rectangular shape and near mirror-image symmetry are indicative of ideal capacitive behaviour^44^,^55^, consistent with the triangular shaped GCD curves at different current densities (Fig. 5b). The maximum specific capacitance of the pseudocapacitors calculated from the GCD curves reaches 291.6 F g^−1^ at 1 A g^−1^, which is very close to the specific capacitance of the AC electrodes (301.3 F g^−1^ at 1 A g^−1^, Supplementary Fig. S9), further indicating the excellent performance of the hybrid electrode. Even at a high current density of 20 A g^−1^, the specific capacitance retains 253.3 F g^−1^, which is 86.9% of the value at 1 A g^−1^, indicating the excellent rate capability. As one of the most important parameters for the practical application of supercapacitors, cycling stability was tested. A high capacitance retention of 92.28% was obtained after 25,000 charge/discharge cycles at a low current density of 2 A g^−1^ (Fig. 5c). The capacitance increases during the initial 600 cycles, which may arise from the activation process^34^. This excellent cycling stability is superior among the supercapacitors based on metal oxides^14^,^18^,^19^,^20^,^21^,^23^,^24^,^26^,^39^,^40^,^49^,^55^,^58^,^60^,^63^,^64^,^65^, indicating the merits of this unique technology. The maximum energy density of the pseudocapacitors was calculated to be 91.13 Wh kg^−1^ at a power density of 750 W kg^−1^ (Fig. 5d). This ultrahigh energy density is higher than the previously reported supercapacitors based on Ni(OH)_2_ and MnO_2_^12^,^20^,^23^,^24^,^26^,^49^,^55^,^57^,^58^,^60^,^62^ and comparable to most of the advanced systems reported in the literature^14^,^16^,^19^,^63^,^64^,^65^. The detailed comparison between our results and the literature data is shown in Supplementary Table S4. Furthermore, at a high power density of 15 kW kg^−1^, high energy density of 79.16 Wh kg^−1^ can still be obtained. Electrochemical impedance spectra were also measured for the pseudocapacitors. The Nyquist plot in Supplementary Fig. S10 shows low equivalent series resistance (0.4 Ω) and charge transfer resistance in the high-frequency region. To demonstrate the capability for large-scale fabrication of this technology and the practical application of the solid-state pseudocapacitors, we assembled two pseudocapacitors with an area of 10 × 10 cm^2^ and connected them in series. After charged to 3 V, the tandem device successfully powered a light-emitting diode logo containing 165 blue lights (Supplementary Fig. S11).

## Discussion

We consider that the unique structure resulting from the metal redox growth mechanism accounts for the outstanding electrochemical performance of the NMNSs. First, vertical alignment and ultrathin thickness of the NMNSs allow easy access of the electrolyte and high-efficiency utilization of the active materials. Second, the alternately arranged small domains in single nanosheets form a large number of interfaces, providing numerous active sites for ion adsorption and insertion, and thus increasing the utilization efficiency of the active materials. Finally, the participation of the substrate element in the redox reaction generates continuous and compact structure at the nanosheets/substrate interfaces, resulting in strong adhesion and thus increasing the operation stability and rate capability. Due to the universality of metal redox reaction we expect that many other hybrid oxides can be prepared by the present strategy, which has been demonstrated by our preliminary research on synthesizing Co(OH)_2_/MnO_2_ and Fe_2_O_3_/MnO_2_ hybrid oxides (Supplementary Figs S12,13).

In summary, we have developed a facile and cost-effective method for large-scale production of NMNSs electrodes by self-reaction of Ni foam substrates in KMnO_4_ solution at room temperature for supercapacitor application. Besides the production simplicity, the electrodes prepared by this method exhibit excellent electrochemical performance. Solid-state asymmetric pseudocapacitors assembled using the electrodes possess ultrahigh energy density and excellent rate capability and cycling stability. This simple metal redox mechanism to synthesize hybrid metal oxides is universally applicable for synthesizing other hybrid oxides and highly promising for not only supercapacitors but also other applications such as catalysis.

## Methods

### Preparation of Ni(OH)_2_/MnO_2_/Ni foam electrodes

Prior to the redox reaction, the Ni foam was cut into pieces of 2 × 4 cm^2^ in size and washed with acetone and deionized water under ultrasonic vibration for 15 min, followed by washing with 0.1 M HCl solution for 5 min. Then, it was thoroughly cleaned with deionized water under ultrasonic vibration and then dried at 60 ^o^C under vacuum. The Ni foam was weighed by an analytical balance (Sartorius BT25S) with a measuring range of 21 g and resolution of 0.01 mg. KMnO_4_ solution (50 mM, 30 ml) was prepared by dissolving the KMnO_4_ powders in deionized water and stirring for 1 h. Subsequently, the cleaned Ni foam was dunked into the KMnO_4_ solution for 24 h at room temperature to grow the hybrid nanosheets. After finishing the growth reaction, the Ni foam was rinsed with deionized water and dried under vacuum and then the Ni(OH)_2_/MnO_2_/Ni foam electrode was obtained. High purity Fe and Co foils (99.9999%) were used to synthesize Co(OH)_2_/MnO_2_ and Fe_2_O_3_/MnO_2_ hybrid oxides by reacting with KMnO_4_ solution following similar procedure.

### Preparation of activated carbon electrodes

The activated carbon electrode was prepared by pressing the mixture (80 wt% activated carbon, 10 wt% carbon black, and 10 wt% polyvinylidene fluoride) on a Ni foam current collector. Subsequently, the electrode was dried at 100 °C.

### Fabrication of solid-state asymmetric pseudocapacitors

The solid-state asymmetric pseudocapacitors were assembled by separating a piece of Ni(OH)_2_/MnO_2_/Ni foam cathode and an activated carbon anode with a separator (Whatman glass fiber filter paper) using PVA/KOH gel as the solid-state electrolyte. The PVA/KOH gel was prepared by mixing PVA (30 g, Mw 89,000–98,000) and KOH (17 g) in deionized water (300 ml) and heating at 90 ^o^C for 1 h under vigorous stirring until the solution became clear. Prior to assembly, the edge of the Ni(OH)_2_/MnO_2_/Ni foam was sanded using sandpaper to expose the pristine metal substrate for connecting the electrode clamp. The electrodes and separator were soaked in the gel for 5 min. Then the electrodes and separator with electrolyte gel were assembled together and solidified at room temperature overnight.

### Material characterization and electrochemical measurement

The structure of the electrode materials was characterized by SEM (FEI Helios Nanolab 600i, 5 kV), TEM/HAADF-STEM (FEI Image Corrected Titan G2 60–300, 300 kV), and XRD (Rigaku D/Max 2500PC, Cu Kα radiation, λ = 1.5406 Å). The bonding states and composition of the samples were determined by XPS (MICROLAB350, VG Scientific Co. Ltd, UK) using a monochromatic Al Kα X-ray source. Raman spectra were recorded using a Renishaw Raman microscope (Renishaw InVia Reflex) with an incident laser wavelength of 514 nm. Electrochemical testing was performed on a CHI 760D electrochemical workstation (Shanghai CH Instrument Company, China). In the three-electrode system, a Pt wire, a Ag/AgCl (3 M KCl) electrode, and a Ni(OH)_2_/MnO_2_/Ni foam were used as the counter, reference, and working electrode, respectively. The edge of the Ni(OH)_2_/MnO_2_/Ni foam was sanded using sandpaper to expose the pristine metal substrate for connecting the electrode clamp. Wanted active area was left to be immersed in the electrolytes for electrochemical testing. The electrolyte solutions were bubbled with Ar for at least 15 min before testing to remove the dissolved O_2_.

### Mass Loading Calculation

The mass increase of the Ni foam after growing the NMNSs is from Mn and O atoms and OH^−^ ions. In brief, according to reaction equation (5), the molar ratio of MnO_2_ to OH^−^ in the hybrid nanosheets is 1:3. Accordingly, from the weight increase of the Ni foam before and after growth reaction, the mass of OH^−^, Ni(OH)_2_, and MnO_2_ in the nanosheets can be obtained. Therefore, by measuring the weight of the Ni foam before and after growth reaction, the total mass of the NMNSs can be calculated.

In detail, *m*(MnO_2_ + OH^−^) = *m*_2_ − *m*_1_, where *m*(MnO_2_ + OH^−^) is the total mass of MnO_2_ and OH^−^, *m*_1_ (g) is the mass of the pristine Ni foam, *m*_2_ (g) is the mass of the Ni(OH)_2_/MnO_2_/Ni foam. Then the mass of Ni(OH)_2_/MnO_2_ composites *m* (g) can be obtained according to the stoichiometric ratio.


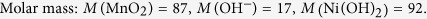



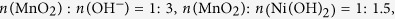



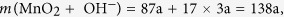



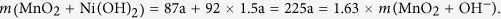


Mass loading: *m*_A_ = *m*(MnO_2_ + Ni(OH)_2_)/*S*, where *S* (cm^2^) is the geometrical area of the Ni foam. *m*_A_ is about 1.2 mg cm^−2^ for the samples after growth reaction for 24 h, which is an average value of ten experiments.

### Electrochemical calculation for three-electrode system

The specific capacitance *C* (F g^−1^) of the NMNSs was calculated from the GCD curves as equation *C* = *It*/*mV*, where *I* (A) is the discharge current, *t* (s) is the discharge time, *V* (V) is the potential change during the discharge, and *m* (g) is the mass of the NMNSs on Ni foam.

### Electrochemical calculation for solid-state asymmetric pseudocapacitors

The specific capacitances *C* (F g^−1^) of the asymmetric pseudocapacitors were calculated from the GCD curves as equation *C* = *It*/*mV*, where *I* (A) is the discharge current, *t* (s) is the discharge time, *V* (V) is the potential change during the discharge, and *m* (g) is the total mass of the active materials in two electrodes. The energy densities *E* (Wh kg^−1^) of the asymmetric pseudocapacitors were calculated from the GCD curves as equation *E* = *CV*^2^/(2 × 3.6), where *C* (F g^−1^) is the specific capacitance of the asymmetric pseudocapacitors, *V* (V) is the potential change during the discharge. The power densities *P* (W kg^−1^) of the asymmetric pseudocapacitors were calculated from the GCD curves as equation *P* = 3,600 × *E*/*t*, where *E* (Wh kg^−1^) is the energy density of the asymmetric pseudocapacitors, *t* (s) is the discharge time.

## Additional Information

**How to cite this article**: Ren, Z. *et al*. Large-scale synthesis of hybrid metal oxides through metal redox mechanism for high-performance pseudocapacitors. *Sci. Rep.* **6**, 20021; doi: 10.1038/srep20021 (2016).

## Supplementary Material

Supplementary Information
