# Supplementary material for: Large-scale synthesis of hybrid metal oxides through metal redox mechanism for high-performance pseudocapacitors
Source: Sci Rep. 2016 Jan 25;6:20021. doi: 10.1038/srep20021 (PMC4726185; doi:10.1038/srep20021)
Supplement: Supplementary Information [file srep20021-s1.pdf]

Supplementary Information

# Large-scale synthesis of hybrid metal oxides through metal redox mechanism for high-performance pseudocapacitors

Zhonghua Ren, Jianpeng Li, Yaqi Ren, Shuguang Wang, Yejun Qiu★, and Jie Yu★

Shenzhen Engineering Lab for Supercapacitor Materials, Shenzhen Key Laboratory for Advanced Materials, Department of Material Science and Engineering, Shenzhen Graduate School, Harbin Institute of Technology, University Town, Shenzhen 518055, China.

★Corresponding author. E-mail: yejunqiu@hitsz.edu.cn; jyu@hitsz.edu.cn

**Table S1.** Standard electrode potentials of various half-reactions (298 K)[1]

| Cathode half-reaction | $E^0$/V | Anode half-reaction | $E^0$/V | $E^0_{cell}$/V |
|---|---|---|---|---|
| $Fe(OH)_2+2e=Fe+2OH^-$ | –0.89 | $MnO_4^-+2H_2O+3e=MnO_2+4OH^-$ | 0.595 | 1.485 |
| $Ni(OH)_2+2e=Ni+2OH^-$ | –0.72 | | | 1.315 |
| $Co(OH)_2+2e=Co+2OH^-$ | –0.73 | | | 1.325 |
| $Mn(OH)_2+2e=Mn+2OH^-$ | –1.55 | | | 2.145 |
| $Zn(OH)_2+2e=Zn+2OH^-$ | –1.245 | | | 1.84 |
| $La(OH)_3+3e=La+3OH^-$ | –2.9 | | | 3.495 |
| $WO_4^{2-}+4H_2O+6e=W+8OH^-$ | –1.01 | | | 1.605 |
| $Bi_2O_3+3H_2O+6e=2Bi+6OH^-$ | –0.46 | | | 1.055 |
| $Cu(OH)_2+2e=Cu+2OH^-$ | –0.222 | | | 0.817 |
| $Cu_2O+H_2O+2e=2Cu+2OH^-$ | –0.36 | | | 0.955 |
| $HSnO_2^-+H_2O+2e=Sn+3OH^-$ | –0.909 | | | 1.504 |
| $PbO+H_2O+2e=Pb+2OH^-$ | –0.58 | | | 1.175 |
| $Ir_2O_3+3H_2O+6e=2Ir+6OH^-$ | 0.098 | | | 0.497 |
| $Pd(OH)_2+2e=Pd+2OH^-$ | 0.07 | | | 0.525 |
| $Pt(OH)_2+2e=Pt+2OH^-$ | 0.14 | | | 0.455 |
| $SbO_2^-+2H_2O+3e=Sb+4OH^-$ | –0.66 | | | 1.255 |
| $SeO_3^{2-}+3H_2O+4e=Se+6OH^-$ | –0.366 | | | 0.961 |
| $Sr(OH)_2+2e=Sr+2OH^-$ | –2.88 | | | 4.436 |
| $Th(OH)_4+4e=Th+4OH^-$ | –2.48 | | | 3.075 |
| $TeO_2^{2-}+3H_2O+4e=Te+6OH^-$ | –0.57 | | | 1.165 |
| $MoO_4^{2-}+4H_2O+6e=Mo+8OH^-$ | –1.05 | | | 1.645 |
| $ReO_4^-+4H_2O+7e=Re+8OH^-$ | –0.584 | | | 1.179 |
| $Sc(OH)_3+3e=Sc+3OH^-$ | –2.6 | | | 3.195 |
| $In(OH)_3+3e= In+3OH^-$ | -0.99 | | | 1.585 |
| $Lu(OH)_3+3e=Lu+3OH^-$ | -2.72 | | | 3.315 |
| $Cr(OH)_3+3e= Cr+3OH^-$ | -1.48 | | | 2.075 |

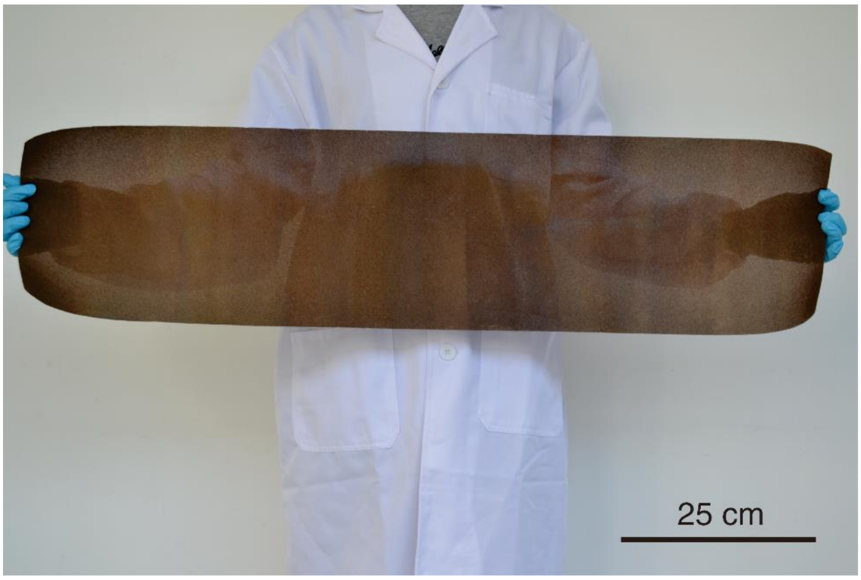


**Figure S1.** A photograph of the Ni foam with an area of 25 × 100 $cm^2$ after redox reaction.

**Table S2.** Previously reported and the present large-area supercapacitor electrodes with different sizes

| Electrode material | Electrode area | Reference |
|---|---|---|
| NMNSs film | 25 × 100 $cm^2$ | This work |
| RGO film | 3 × 8.7 $cm^2$ | 2 |
| PEDOT:PSS film | 18 × 18 $cm^2$ | 3 |
| Core-shell graphene/porous carbon woven fabric film | 10-cm wide | 4 |
| $MnO_2$ nanorod arrays | 4 × 4 $cm^2$ | 5 |
| $Co_xNi_yAl_z$LTHs | 3×4 $cm^2$ | 6 |
| Carbon cloth/$Fe_2O_3$ | 14.50 × 31.00 $cm^2$ | 7 |
| $Cu_2O$@Cu nanoneedle arrays | 5 × 5 $cm^2$ | 8 |
| Graphene oxide film | 200 $cm^2$ | 9 |

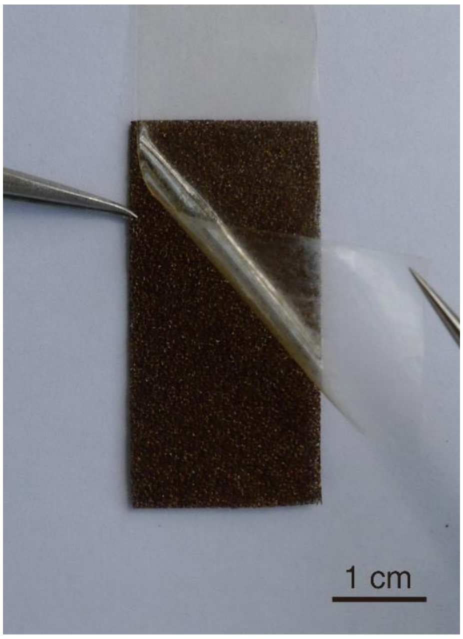


**Figure S2.** An optical image showing the adhesion test process of the nanosheet films. The nanosheet film keeps intact after peeling a 3M scotch tape sticking on it, where no color changes are observed for both the film and the peeled tape, indicating that the anchoring strength of the grown nanosheets on the substrate is very high.

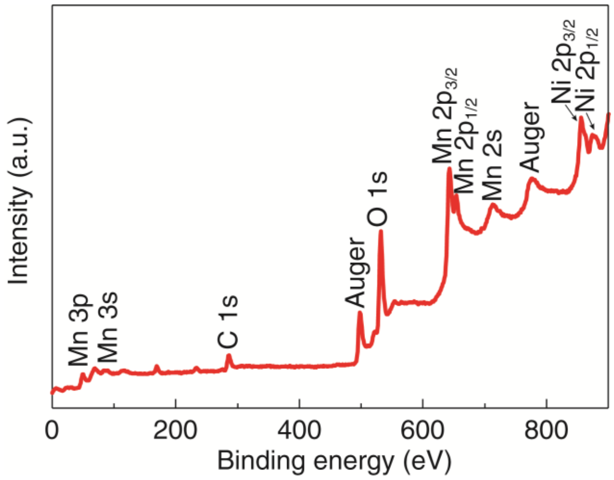


**Figure S3.** XPS survey spectrum of the $Ni(OH)_2/MnO_2$ hybrid nanosheets.

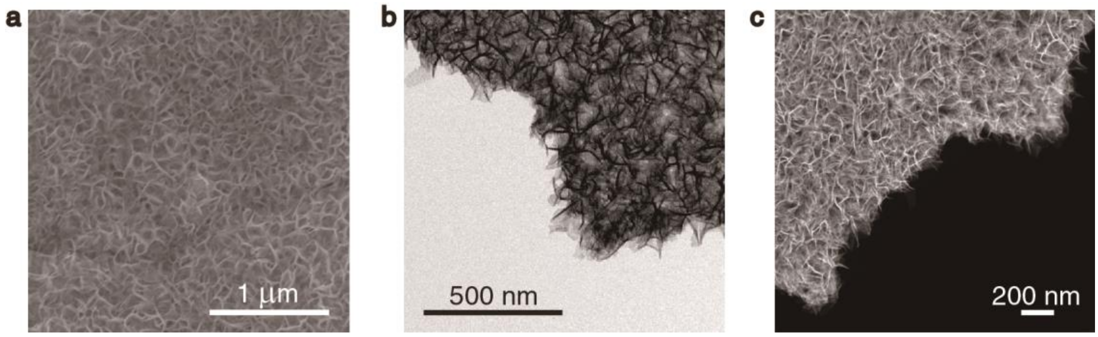


**Figure S4.** Morphology of the obtained $Ni(OH)_2/MnO_2$ hybrid nanosheets. (a) SEM image. (b) transmission electron microscopy (TEM) image. (c) High-angle annular dark field scanning transmission electron microscopy (HAADF-STEM) image.

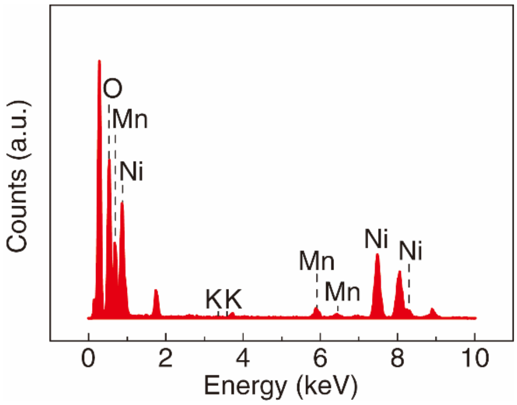


**Figure S5.** EDX spectrum of a single $Ni(OH)_2/MnO_2$ hybrid nanosheet. The K element should come from $KMnO_4$.

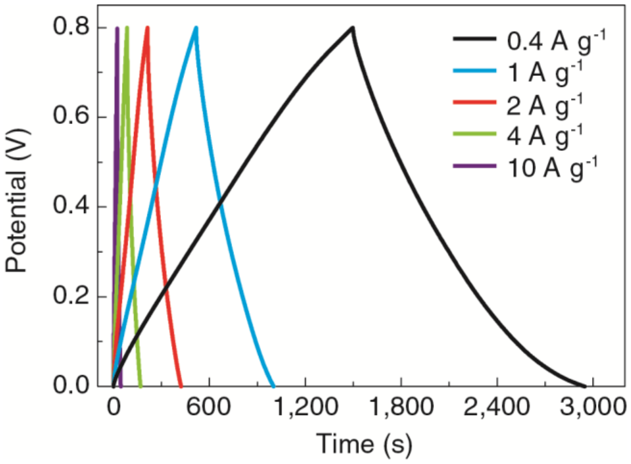


**Figure S6.** GCD curves of the hybrid nanosheets at different current densities in pure 1 M $Na_2SO_4$ solution.

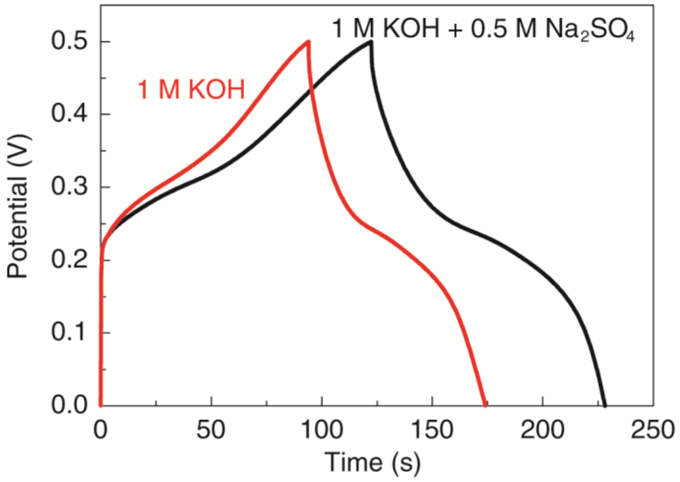


**Figure S7.** GCD curves of the $Ni(OH)_2/MnO_2$ nanosheets in different electrolytes at a current density of 10 A $g^{-1}$. The nanosheets have a longer discharge time in the mixed electrolyte (1 M KOH + 0.5 M $Na_2SO_4$) than in pure 1 M KOH.

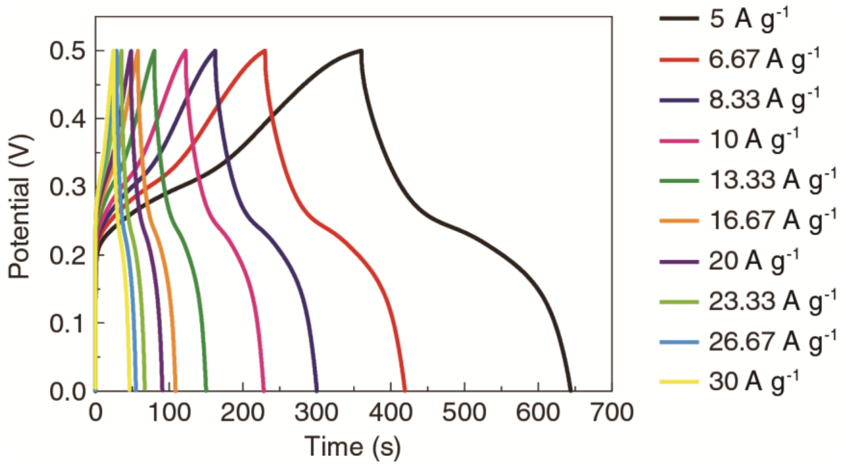


**Figure S8.** GCD curves of the $Ni(OH)_2/MnO_2$ nanosheets in the mixed electrolyte of 1 M KOH + 0.5 M $Na_2SO_4$ at different current densities.

**Table S3.** Electrochemical parameters of the electrode materials of the present work and previously reported based on $Ni(OH)_2$ and $MnO_2$

| Electrode material | Specific capacitance | Current density/Scan rate | Electrolyte | Potential range | Reference |
|---|---|---|---|---|---|
| NMNSs | 2,937 F $g^{-1}$<br><br>2,325 F $g^{-1}$<br>723 F $g^{-1}$ | 5 A $g^{-1}$<br><br>5 A $g^{-1}$<br>0.4 A $g^{-1}$ | 1 M KOH + 0.5 M $Na_2SO_4$<br>1 M KOH<br>1 M $Na_2SO_4$ | 0~0.5 V<br><br>0~0.5 V<br>0~0.8 V | This work |
| $MnO_2$ on Pt foil | 1,380 F $g^{-1}$ | 5 mV $s^{-1}$ | 0.1 M $Na_2SO_4$ | 0~0.9 V | 10 |
| α-$Ni(OH)_2$ on Ni foam | 4,172.5 F $g^{-1}$ | 1 A $g^{-1}$ | 6 M KOH | -0.05~0.35 V | 11 |
| $Ni(OH)_2$/UGF | 166 F $g^{-1}$ | 0.5 A $g^{-1}$ | 6 M KOH | 0~0.5 V | 12 |
| $Co_3O_4$@$MnO_2$ on stainless steel | 480 F $g^{-1}$ | 2.67 A $g^{-1}$ | 1 M LiOH | -0.2~0.6 V | 13 |
| $Ni(OH)_2$-$MnO_2$ on Ni foam | 2,628 F $g^{-1}$ | 3 A $g^{-1}$ | 1 M KOH | 0~0.5 V | 14 |
| RGO-$Ni(OH)_2$ on Ni foam | 1,717 F $g^{-1}$ | 0.5 A $g^{-1}$ | 2 M KOH | 0~0.38 V | 15 |
| Ni-Co LDH on Ni foam | 2,682 F $g^{-1}$ | 3 A $g^{-1}$ | 1 M KOH | 0~0.5 V | 16 |

| | | | | | |
|---|---|---|---|---|---|
| Amorphous $Ni(OH)_2$ on graphite sheet | 2,188 F $g^{-1}$ | 1 mV $s^{-1}$ | 1 M KOH | 0~0.5 V | 17 |
| $Ni(OH)_2$/graphene on Ni foam | 1,735 F $g^{-1}$ | 1 mV $s^{-1}$ | 6 M KOH | -0.1~0.45 V | 18 |
| $WO_{3-x}$@Au@$MnO_2$ on carbon fabric | 588 F $g^{-1}$ | 10 mV $s^{-1}$ | 0.1 M $Na_2SO_4$ | 0~0.8 V | 19 |
| $Ni(OH)_2$/CNT/NF | 3,300 F $g^{-1}$ | 2.5 mA $cm^{-2}$ | KOH | 0~0.4 V | 20 |
| $Ni(OH)_2$ on Ni foil | 1,765 F $g^{-1}$ | 2 mV $s^{-1}$ | 6 M KOH | 0~0.475 V | 21 |
| MWCNT/amor-$Ni(OH)_2$/PEDOT:PSS on carbon cloth | 3,262 F $g^{-1}$ | 5 mV $s^{-1}$ | 1 M KOH | 0~0.5 V | 22 |
| $Ni(OH)_2$-$MnO_2$ | 355 F $g^{-1}$<br>487.4 F $g^{-1}$ | 0.5 A $g^{-1}$<br>1 A $g^{-1}$ | 1 M $Na_2SO_4$<br>1 M KOH | -0.1~0.9 V<br>-0.2~0.5 V | 23 |
| $Ni(OH)_2$-$MnO_2$-RGO on Ni foam | 1,985 F $g^{-1}$ | 2 A $g^{-1}$ | 1 M KOH | 0~0.5 V | 24 |
| $MnO_2$/RGO/$Ni(OH)_2$ on Ni foam | 3,296.9 F $g^{-1}$ | 1.3 A $g^{-1}$ | 1 M KOH | -0.1~0.5 V | 25 |
| *p*-BC@$MnO_2$ | 254.64 F $g^{-1}$ | 1 A $g^{-1}$ | 1 M $Na_2SO_4$ | 0~1 V | 26 |

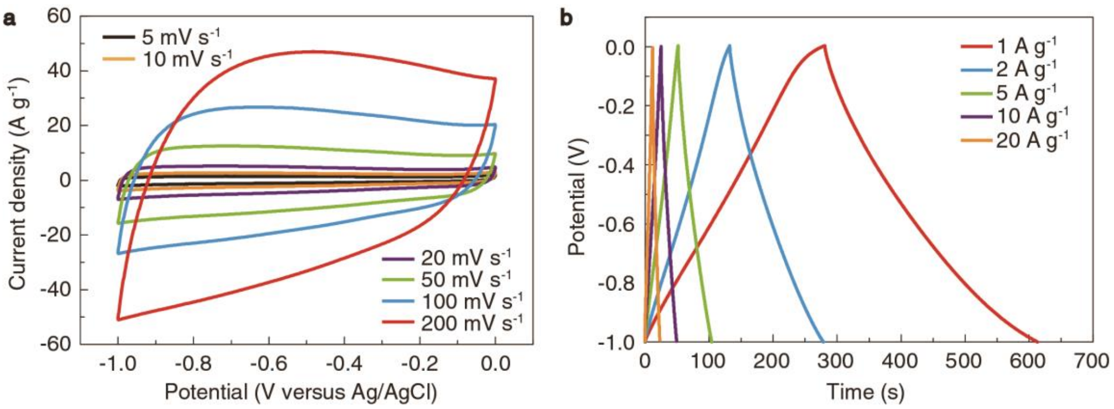


**Figure S9.** Electrochemical characterization of activated carbon in KOH electrolyte. (a) CV curves at different scan rates. (b) GCD curves at different current densities.

**Table S4.** Electrochemical parameters of supercapacitors of the present work and previously reported based on oxides

| Electrode materials | Specific capacitance | Current density/Scan rate | Electrolyte | Energy density | Power density | Capacitance retention after cycles | Reference |
|---|---|---|---|---|---|---|---|
| NMNSs//AC | 291.6 F $g^{-1}$ | 1 A $g^{-1}$ | PVA/KOH gel | 91.13 Wh $kg^{-1}$ | 750 W $kg^{-1}$ | 92.28% after 25,000 cycles | This work |
| $Ni(OH)_2$/UGF//a-MEGO | 119 F $g^{-1}$ | 1 A $g^{-1}$ | 6 M KOH | 13.4 Wh $kg^{-1}$ | / | 63.2% after 10,000 cycles | 12 |
| β-$Co(OH)_2$//N-doped graphene | 241.9 F $g^{-1}$ | 1 A $g^{-1}$ | PVA/KOH gel | 98.9 Wh $kg^{-1}$ | 17,981 W $kg^{-1}$ | 93.2% after 10,000 cycles | 27 |
| NiOOH/$Ni_3S_2$/3D-G//$Fe_3O_4$/rGO | 233 F $g^{-1}$ | 5 mV $s^{-1}$ | 1 M KOH | 82.5 Wh $kg^{-1}$ | 930 W $kg^{-1}$ | 26% after 3,000 cycles | 28 |
| $Ni(OH)_2$-$MnO_2$//RGO | 538 F $g^{-1}$ | 1.4 A $g^{-1}$ | 1 M KOH | 186 Wh $kg^{-1}$ | 778 W $kg^{-1}$ | 76% after 3,000 cycles | 14 |
| $Co_3O_4$-H//$Co_3O_4$-H | 580 F $g^{-1}$ | 1 A $g^{-1}$ | PVA/KOH gel | 80 Wh $kg^{-1}$ | 500 W $kg^{-1}$ | 86.3% after 20,000 cycles | 29 |
| RGO-$Ni(OH)_2$//RGO | 210.9 F $g^{-1}$ | 1 A $g^{-1}$ | 2 M KOH | 75 Wh $kg^{-1}$ | 800 W $kg^{-1}$ | 89% after 10,000 cycles | 15 |
| Ni-Co LDH//RGO | / | / | 1 M KOH | 188 Wh $kg^{-1}$ | 1,499 W $kg^{-1}$ | 82% after 5,000 cycles | 16 |
| Amorphous $Ni(OH)_2$//AC | 153 F $g^{-1}$ | 5 mV $s^{-1}$ | 1 M KOH | 35.7 Wh $kg^{-1}$ | 490 W $kg^{-1}$ | 81% after 10,000 cycles | 17 |
| graphene/$MnO_2$//SWNTs | / | / | 0.5 M $Na_2SO_4$ | 12.5 Wh $kg^{-1}$ | / | 95% after 5,000 cycles | 30 |
| $Ni(OH)_2$/graphene//graphene | 218.4 F $g^{-1}$ | 1 mV $s^{-1}$ | 6 M KOH | 77.8 Wh $kg^{-1}$ | 174.7 W $kg^{-1}$ | 94.3% after 3,000 cycles | 18 |
| $MnO_2$/CNF//$Bi_2O_3$/CNF | 25.2 F $g^{-1}$ | 1.5 mA $cm^{-2}$ | 1 M $Na_2SO_4$ | 11.3 Wh $kg^{-1}$ | 352.6 W $kg^{-1}$ | 85% after 4,000 cycles | 31 |
| CuO//AC | 72.4 F $g^{-1}$ | 1 A $g^{-1}$ | 3 M KOH | 19.7 Wh $kg^{-1}$ | 700 W $kg^{-1}$ | 96% after 3,000 cycles | 32 |
| $MnO_2$/graphene//graphene | / | / | 1 M $Na_2SO_4$ | 30.4 Wh $kg^{-1}$ | / | 79% after 1,000 cycles | 33 |
| $Ni(OH)_2$/CNT/NF//AC | 112.5 F $g^{-1}$ | 2.5 mA $cm^{-2}$ | KOH | 50.6 Wh $kg^{-1}$ | 95 W $kg^{-1}$ | 83% after 3,000 cycles | 20 |
| $Ni(OH)_2$//AC | 192 F $g^{-1}$ | 0.9 A $g^{-1}$ | 6 M KOH | 68 Wh | 200 W | 90% after | 21 |

| | | | | | | | |
|---|---|---|---|---|---|---|---|
| | | | | kg$^{-1}$ | kg$^{-1}$ | 10,000 cycles | |
| MWCNT/amor-$Ni(OH)_2$/PEDOT:PSS//rGO/CNT | 179.8 F g$^{-1}$ | 1 A g$^{-1}$ | 1 M KOH | 58.5 Wh kg$^{-1}$ | 780 W kg$^{-1}$ | 86% after 30,000 cycles | 22 |
| $Ni(OH)_2$-$MnO_2$-RGO//FRGO | / | / | 1 M KOH | 32.6 Wh kg$^{-1}$ | 305 W kg$^{-1}$ | 75% after 2,000 cycles | 24 |
| *p*-BC@$MnO_2$//*p*-BC/N | 254.64 F g$^{-1}$ | 1 A g$^{-1}$ | 1 M $Na_2SO_4$ | 32.91 Wh kg$^{-1}$ | 250 W kg$^{-1}$ | 95.4% after 2,000 cycles | 26 |
| NiO-3D graphene//AC | 319 F g$^{-1}$ | 1 mV s$^{-1}$ | 1 M NaOH | 138 Wh kg$^{-1}$ | 5,250 W kg$^{-1}$ | 85% after 5,000 cycles | 34 |
| $NiMoO_4$//AC | 151.7 F g$^{-1}$ | 1 A g$^{-1}$ | 2 M KOH | 60.9 Wh kg$^{-1}$ | 850 W kg$^{-1}$ | 85.7% after 10,000 cycles | 35 |
| GF-CNT@$Fe_2O_3$//GF-$CoMoO_4$ | 210 F g$^{-1}$ | 1.5 A g$^{-1}$ | 2 M KOH | 74.7 Wh kg$^{-1}$ | 1400 W kg$^{-1}$ | 95.4% after 50,000 cycles | 36 |

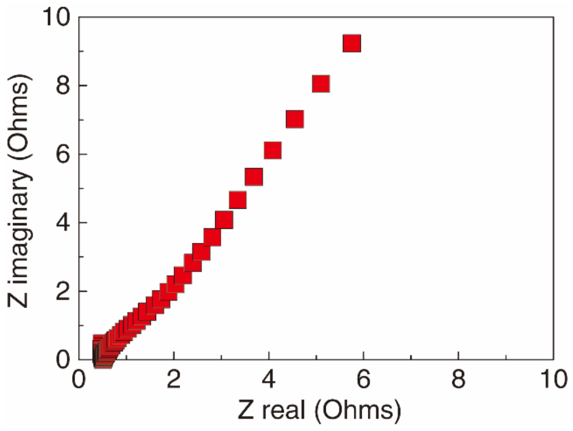


**Figure S10.** A Nyquist plot for the asymmetric pseudocapacitors.

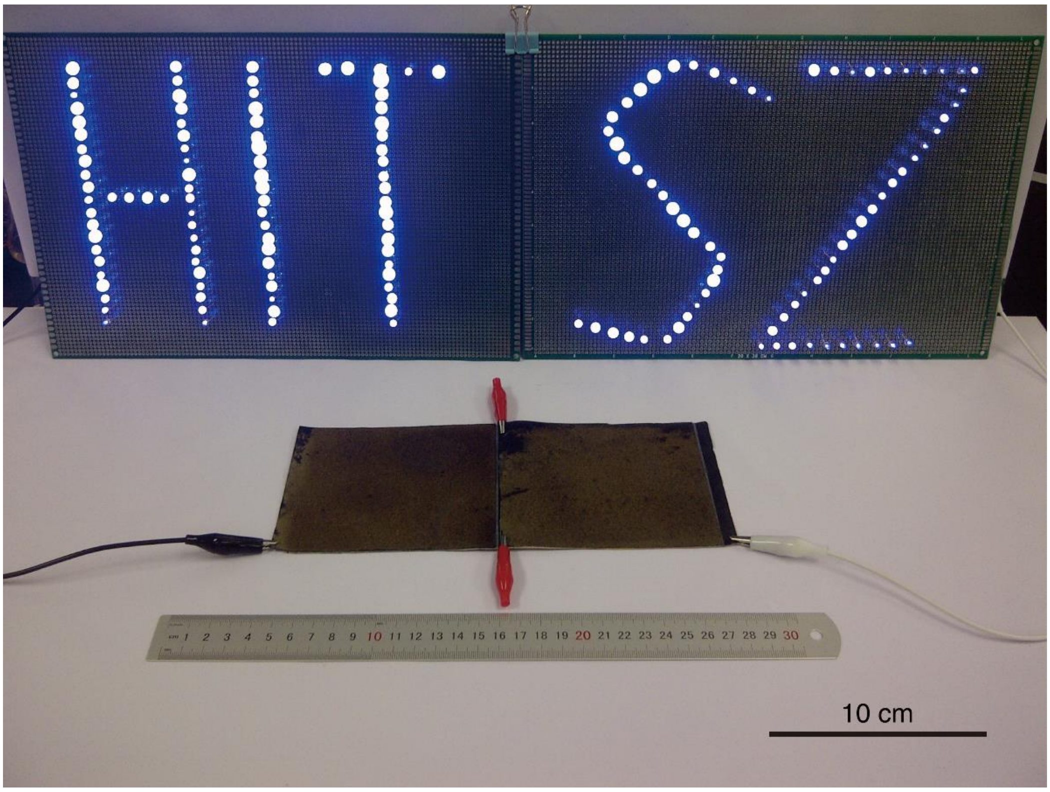


**Figure S11.** An optical image of LED logo containing 165 blue lights powered by the tandem solid-state devices with an area of 10 × 10 $cm^2$.

According to Supplementary Table S1 the reactions employing metal Co and Fe to reduce $MnO_4^-$ to produce the Co/Mn and Fe/Mn hybrid oxides are thermodynamically favored. We soaked Co and Fe foils in $KMnO_4$ solution for 24 h. It was found that the colour of the Co and Fe foils changed (Supplementary Figs. S12,13), suggesting formation of new substances. After characterization by XRD and Raman the products grown on the Co and Fe foils were determined to be $Co(OH)_2/MnO_2$ and $Fe_2O_3/MnO_2$ (Supplementary Figs. S12,13), respectively. These results strongly support that the metal redox mechanism is a universally applicable method to synthesize hybrid metal oxides.

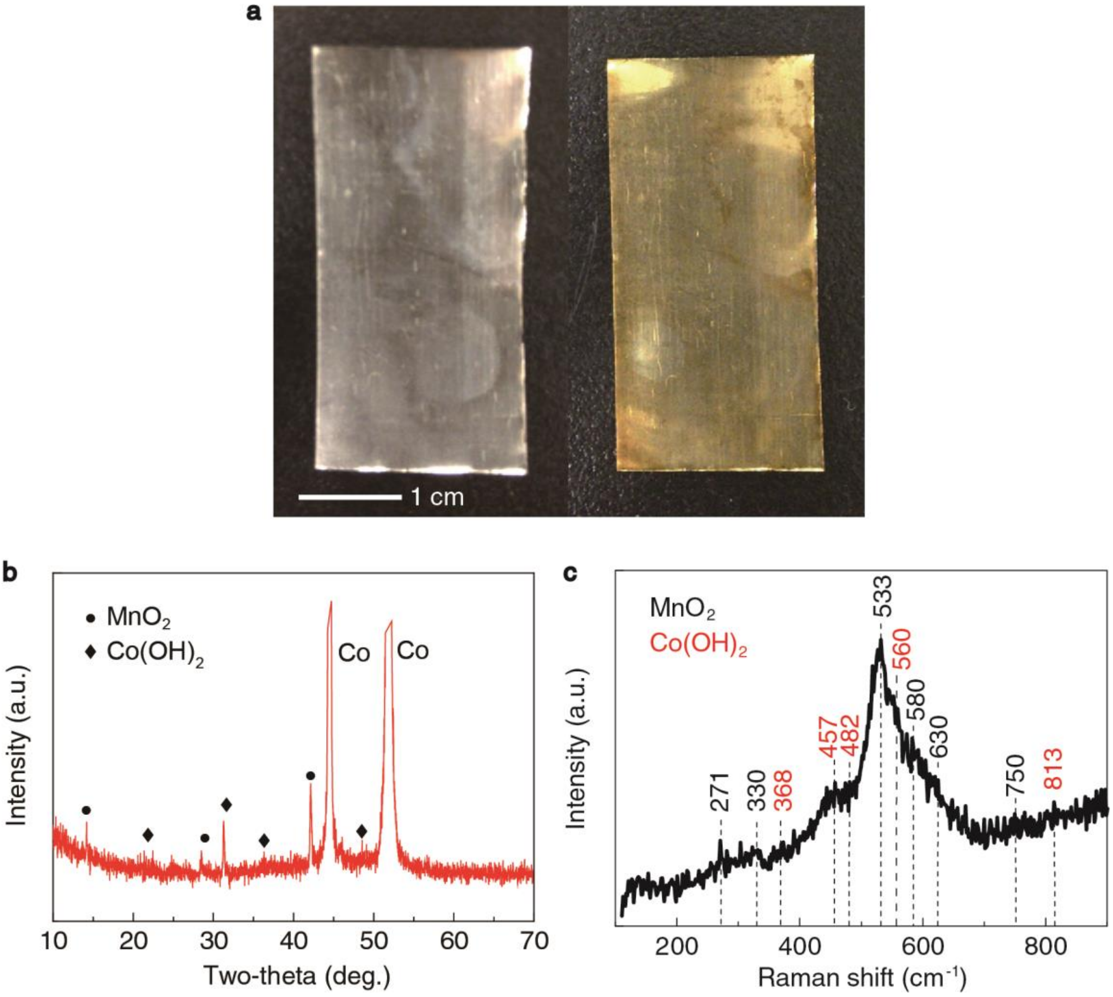


**Figure S12.** (a) An optical image of the Co foil before and after growth reaction. (b–c) A typical XRD pattern (b) and Raman spectrum (c) of the $Co(OH)_2/MnO_2$ hybrids.

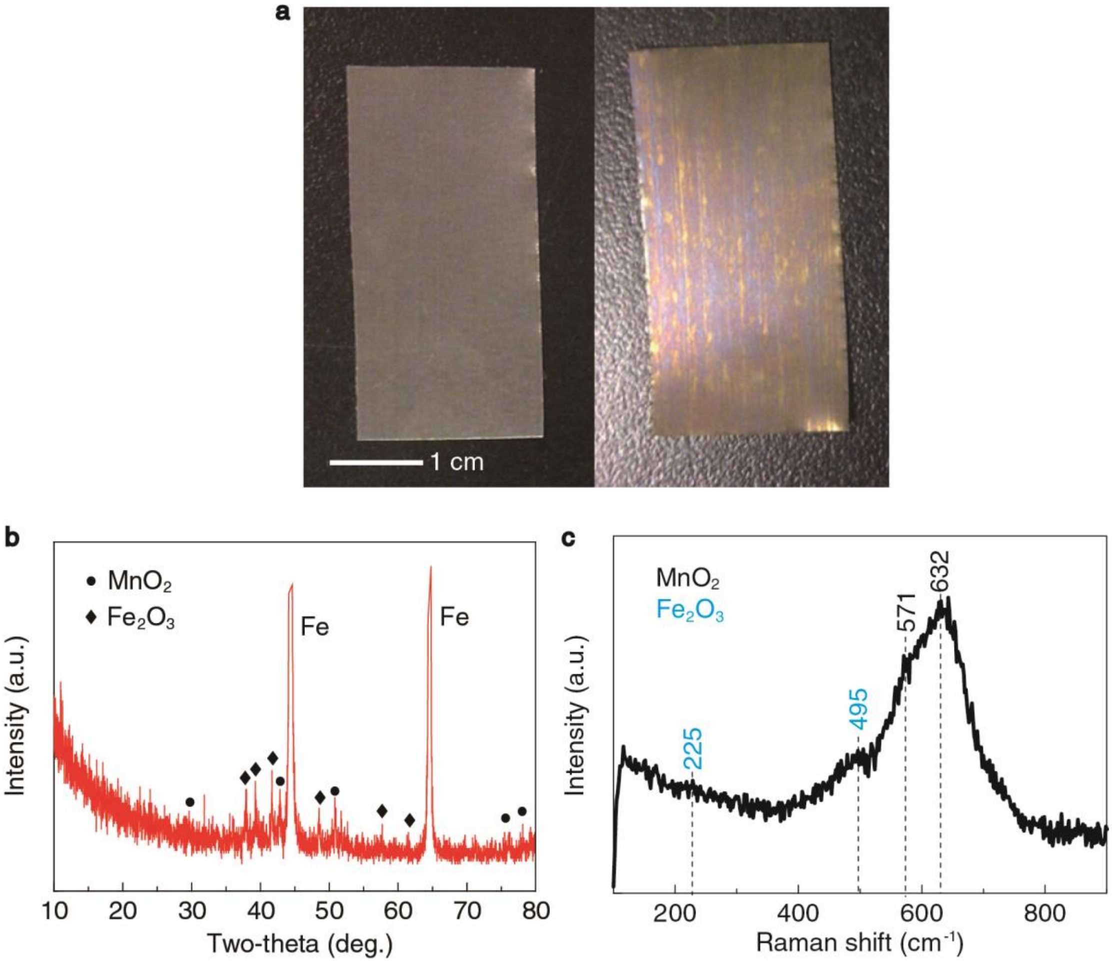


**Figure S13.** (a) An optical image of the Fe foil before and after growth reaction. (b–c) A typical XRD pattern (b) and Raman spectrum (c) of the $Fe_2O_3$/$MnO_2$ hybrids.

## Supplementary References


1 Lide, D. R. *Handbook of Chemistry and Physics*, 8-23–8-32 (CRC Press LLC, Boca Raton, 2004).

2 Xiong, Z., Liao, C., Han, W. & Wang, X. Mechanically tough large-area hierarchical porous graphene films for high-performance flexible supercapacitor applications. *Adv. Mater.* **27,** 4469–4475 (2015).

3 Higgins, T. M. & Coleman, J. N. Avoiding resistance limitations in high-performance transparent supercapacitor electrodes based on large-area, high-conductivity PEDOT:PSS films. *ACS Appl. Mater. Interfaces* **7,** 16495−16506 (2015).

4 Li, X. *et al*. Large-area flexible core-shell graphene/porous carbon woven fabric films for fiber supercapacitor electrodes. *Adv. Funct. Mater.* **23,** 4862–4869 (2013).

5 Lu, X. *et al*. Facile synthesis of large-area manganese oxide nanorod arrays as a high-performance electrochemical supercapacitor. *Energy Environ. Sci.* **4,** 2915–2921 (2011).

6 Gupta, V., Gupta, S. & Miura, N. Electrochemically synthesized large area network of $Co_xNi_yAl_z$ layered triple hydroxides nanosheets: a high performance supercapacitor. *J. Power Sources* **189,** 1292–1295 (2009).

7 Chen, L.-F., Yu, Z.-Y., Ma, X., Li, Z.-Y. & Yu, S.-H. *In situ* hydrothermal growth of ferric oxides on carbon cloth for low-cost and scalable high-energy-density supercapacitors. *Nano Energy* **9,** 345–354 (2014).

8 Dong, C. *et al*. 3D binder-free $Cu_2O$@Cu nanoneedle arrays for high-performance asymmetric supercapacitors. *J. Mater. Chem. A* **2,** 18229–18235 (2014).

9 Zhang, M., Huang, L., Chen, J., Li, C. & Shi, G. Ultratough, ultrastrong, and highly conductive graphene films with arbitrary sizes. *Adv. Mater.* **26,** 7588−7592 (2014).

10 Toupin, M., Brousse, T. & Belanger, D. Charge storage mechanism of $MnO_2$ electrode used in aqueous electrochemical capacitor. *Chem. Mater.* **16,** 3184–3190 (2004).

11 Zhu, Y. *et al*. Ultrathin nickel hydroxide and oxide nanosheets: synthesis, characterizations and excellent supercapacitor performances. *Sci. Rep*. **4,** 5787 (2014).

12 Ji, J. *et al*. Nanoporous $Ni(OH)_2$ thin film on 3D ultrathin-graphite foam for asymmetric supercapacitor. *ACS Nano* **7,** 6237−6243 (2013).

13 Liu, J. *et al.* $Co_3O_4$ nanowire@$MnO_2$ ultrathin nanosheet core/shell arrays: a new class of high-performance pseudocapacitive materials. *Adv. Mater.* **23,** 2076–2081 (2011).
14 Chen, H. *et al.* One-step fabrication of ultrathin porous nickel hydroxide-manganese dioxide hybrid nanosheets for supercapacitor electrodes with excellent capacitive performance. *Adv. Energy Mater.* **3,** 1636–1646 (2013).
15 Liu, Y., Wang, R. & Yan, X. Ultra-small nickel hydroxide nanoparticles and reduced graphene oxide sheets for the application in high-performance asymmetric supercapacitor. *Sci. Rep.* **5,** 11095 (2015).
16 Chen, H., Hu, L., Chen, M., Yan, Y. & Wu, L. Nickel-cobalt layered double hydroxide nanosheets for high-performance supercapacitor electrode materials. *Adv. Funct. Mater.* **24,** 934–942 (2014).
17 Li, H. B. *et al.* Amorphous nickel hydroxide nanospheres with ultrahigh capacitance and energy density as electrochemical pseudocapacitor materials. *Nat. Commun.* **4,** 1894 (2013).
18 Yan, J. *et al.* Advanced asymmetric supercapacitors based on $Ni(OH)_2$/graphene and porous graphene electrodes with high energy density. *Adv. Funct. Mater.* **22,** 2632–2641 (2012).
19 Lu, X. *et al.* $WO_{3-x}$@Au@$MnO_2$ core-shell nanowires on carbon fabric for high-performance flexible supercapacitors. *Adv. Mater.* **24,** 938–944 (2012).
20 Tang, Z., Tang, C.-h. & Gong, H. A high energy density asymmetric supercapacitor from nano-architectured $Ni(OH)_2$/carbon nanotube electrodes. *Adv. Funct. Mater.* **22,** 1272–1278 (2012).
21 Yang, Y. *et al.* Hydrothermally formed three-dimensional nanoporous $Ni(OH)_2$ thin-film supercapacitors. *ACS Nano* **8,** 9622–9628 (2014).
22 Jiang, W. *et al.* Ternary hybrids of amorphous nickel hydroxide–carbon nanotube-conducting polymer for supercapacitors with high energy density, excellent rate capability, and long cycle life. *Adv. Funct. Mater.* **25,** 1063–1073 (2015).
23 Jiang, H., Li, C., Sun, T. & Ma, J. High-performance supercapacitor material based on $Ni(OH)_2$ nanowire-$MnO_2$ nanoflakes core-shell nanostructures. *Chem. Commun.* **48,** 2606–2608 (2012).
24 Chen, H., Zhou, S. & Wu, L. Porous nickel hydroxide-manganese dioxide-reduced graphene oxide ternary hybrid spheres as excellent supercapacitor electrode materials. *ACS Appl. Mater. Interfaces* **6,** 8621−8630 (2014).

25 Min, S. *et al*. Hydrothermal growth of $MnO_2$/RGO/Ni$(OH)_2$ on nickel foam with superior supercapacitor performance. *RSC Adv*. **5,** 62571−62576 (2015).
26 Chen, L.-F., Huang, Z.-H., Liang, H.-W., Guan, Q.-F. & Yu, S.-H. Bacterial-cellulose-derived carbon nanofiber@$MnO_2$ and nitrogen-doped carbon nanofiber electrode materials: an asymmetric supercapacitor with high energy and power density. *Adv. Mater.* **25,** 4746–4752 (2013).
27 Gao, S. *et al*. Ultrahigh energy density realized by a single-layer β-Co$(OH)_2$ all-solid-state asymmetric supercapacitor. *Angew. Chem. Int. Ed.* **53,** 12789–12793 (2014).
28 Lin, T.-W., Dai, C.-S. & Hung, K.-C. High energy density asymmetric supercapacitor based on NiOOH/$Ni_3S_2$/3D graphene and $Fe_3O_4$/graphene composite electrodes. *Sci. Rep*. **4,** 7274 (2014).
29 Liao, Q., Li, N., Jin, S., Yang, G. & Wang, C. All-solid-state symmetric supercapacitor based on $Co_3O_4$ nanoparticles on vertically aligned graphene. *ACS Nano* **9,** 5310–5317 (2015).
30 Yu, G. *et al*. Solution-processed graphene/$MnO_2$ nanostructured textiles for high-performance electrochemical capacitors. *Nano Lett.* **11,** 2905–2911 (2011).
31 Xu, H. *et al*. Flexible asymmetric micro-supercapacitors based on $Bi_2O_3$ and $MnO_2$ nanoflowers: larger areal mass promises higher energy density. *Adv. Energy Mater.* **5,** 1401882 (2015).
32 Moosavifard, S. E. *et al*. Designing 3D highly ordered nanoporous CuO electrodes for high-performance asymmetric supercapacitors. *ACS Appl. Mater. Interfaces* **7,** 4851−4860 (2015).
33 Wu, Z.-S. *et al*. High-energy $MnO_2$ nanowire/graphene and graphene asymmetric electrochemical capacitors. *ACS Nano* **4,** 5835–5842 (2010).
34 Wang, C. *et al*. Hierarchical composite electrodes of nickel oxide nanoflake 3D graphene for high-performance pseudocapacitors. *Adv. Funct. Mater.* **24,** 6372–6380 (2014).
35 Peng, S., Li, L., Wu, H. B., Madhavi, S. & Lou, X. W. (David). Controlled growth of $NiMoO_4$ nanosheet and nanorod arrays on various conductive substrates as advanced electrodes for asymmetric supercapacitors. *Adv. Energy Mater.* **5,** 1401172 (2015).
36 Guan, C. *et al*. Iron oxide-decorated carbon for supercapacitor anodes with ultrahigh energy density and outstanding cycling stability. *ACS Nano* **9,** 5198–5207 (2015).
